# Biobank Quality Management in the BBMRI.be Network

**DOI:** 10.3389/fmed.2019.00141

**Published:** 2019-06-26

**Authors:** Loes Linsen, Veronique T'Joen, Catherine Van Der Straeten, Kristel Van Landuyt, Etienne Marbaix, Sofie Bekaert, Nadine Ectors

**Affiliations:** ^1^Biobank, University Hospitals Leuven, Leuven, Belgium; ^2^Bioresource Center Ghent, Health Innovation and Research Institute, University Hospital Ghent, Ghent, Belgium; ^3^Pathology Department, de Duve Institute, Saint-Luc University Clinics, Catholic University of Louvain, Brussels, Belgium; ^4^Department of Public Health and Primary Care, Faculty for Medicine and Health Sciences, Ghent University, Ghent, Belgium

**Keywords:** biobank, quality, survey, audit, BBMRI.be, ISO 20387, QMS

## Abstract

From as early as 2005, different guidelines and quality standards covering biobank activities and sample handling methods have been developed to improve and guarantee the reproducibility of biomarker research. Ten years on, the BBMRI.be Quality working group wanted to gauge the current situation of these aspects in the biobanks of the BBMRI.be network. To this end, two online surveys were launched (fall 2017 and fall 2018) to the biobank quality managers in the BBMRI.be network to determine the status and setup of their current quality management system (QMS) and how their QMS and related practices have evolved over a 14 month time period. All biobanks addressed by the two surveys provided a complete response (12 and 13, respectively). A QMS was implemented in 85% of biobanks, with 4 standards emerging as primary basis. Supplementary guidelines were used, with a strong preference for the ISBER best practices for biobanks. The Standard Preanalytical Code—an indicator of the preanalytical lifecycle of a biospecimen impacting the downstream analysis results—was already implemented in 50% of the biobanks while the other half intends future implementation. To assess and maintain the quality of their QMS, 62% of biobanks used self-assessment tools and 71% participated in proficiency testing schemes. The majority of biobanks had implemented procedures for general and biobank specific activities. However, policies regarding the business and sustainability aspect of biobank were only implemented in a limited number of biobanks. A clear desire for a peer-review audit was expressed by 69% of biobanks, with over half of them intending to implement the recently published biobank standard ISO20387. Overall, the biobanks of the BBMRI.be network have actively implemented a solid quality approach in their practices. The implementation of ISO 20387 may bring further professionalization of activities. Based on the needs expressed in this survey, the Quality working group will be setting up an audit program for the BBMRI.be biobanks, to enhance, harmonize and streamline their activities. On the whole, the biobanks in the BBMRI.be network are able to substantially contribute to translational research, as a primary facilitator guaranteeing high quality standards and reproducibility.

## Introduction

Irreproducibility of results has been identified as a major undermining factor for translating research results into clinical applications (1). Different categories of errors contribute to this irreproducibility, with biological reagents and reference materials having the biggest impact (2). It has also been shown that standardization and auditing of biological materials—through biological resource centers or biobanks—can enhance cumulative production of scientific knowledge by improving both availability and reliability of research inputs (3). This need for biospecimen handling standards and the professionalization of biobanking practices to improve research outcome was recognized more than a decade ago. As early as 2005, the “International Society for Biological and Environmental Repositories” (ISBER) published the first version of their best practices in order to support the increasing demands for specific high quality biological material (4). Concurrently, different organizations, biobank networks and national initiatives all worked on best practices and guidelines to address the need for more professionalized biobanking practices and quality management systems (QMS) (5–9). Additionally, significant effort has been put in creating technical standards for pre-examination processes such as those developed within the SPIDIA project (10, 11), for capturing pre-analytical factors such as the Standard Pre-analytical Code (12, 13) and standardized data collection (14–16) to allow fit-for-purpose biological sample management. Finally, educational programs for biobank personnel have been set-up to further professionalize the discipline (17–19).

At the same time, three biobank networks were established in Belgium: the Flemish Biobank Network (FBN) [formerly known as the Center for Medical Innovation (CMI)], the Belgian Virtual Tumorbiobank (BVT), and the Biothèque Wallonie Bruxelles (BWB). A common goal of these networks is to improve and/or harmonize the quality of the biospecimens for the purpose of high-quality collaborative research, albeit through a different approach. The FBN was initiated in 2010 by the Center for Medical Innovation (CMI, Flemish government). The CMI was established to stimulate translational biomedical research and to reach a significant economic value in Flanders by setting up 4 clinical research centers within the Flemish universities and university hospitals. The initial focus lay on advancing biobank professionalization and harmonization within Flanders for five focus disease domains (inflammatory bowel disease, rheumatoid arthritis, diabetes type I, sudden cardiac death and hepatological/hepatotropic diseases). Apart from defining local ethical and legal guidelines, a key result of the CMI initiative was the publication and implementation of the uniform CMI biobank quality guidelines. These were based on the ISO 9001:2008 standard and the OECD guidelines for biorepositories and allowed standardization within the Flemish biobanks. All biobanks of the network were peer-review audited in 2014 according to the CMI quality guidelines. In parallel, a minimal data set of 14 attributes was defined to enable the setup of a centralized virtual catalog for sample query to facilitate collaboration within the five focus domains. In analogy to the FBN network, the BWB project was set up in 2012, incorporating the academic biobanks in Wallonia and Brussels, with the objective of providing a virtual catalog of biospecimens to facilitate translational research. The BWB also established QMS guidelines for biobanks, initially based on the guidelines previously defined by the FBN, which have been used by the BWB biobanks as a basis for their QMS. The BVT was created in 2008 as part of the Belgian National Cancer Plan, which intended to fight cancer by integrating all aspects of the fight against the disease. The aim of the BVT is to centralize standardized and curated data of available residual human tumor samples, collected in the university hospitals and liaised laboratories, in an easy lookup tool. The pseudonymized database is accessible for researchers to query and trace their samples of interest to the local biobanks of the network, where the samples can be released for research projects. Additionally, the BVT also strives to optimize quality by creating awareness about data quality and sample collection by incorporating these elements in the requested standardized dataset.

Since 2013, these three biobank networks are participating in BBMRI.be, the national node of the European Research Infrastructure BBMRI-ERIC, effectively gathering 13 biobanks within one network. Within this national node of BBMRI.be, a Quality working group was established with the aim to define a consensus approach to harmonized QMS systems, biobank sample flows and procedures based upon existing international, European, Belgian and regional requirements. Ten years after the start-up of the Belgian biobank networks, the BBMRI.be Quality working group wanted to gauge the current quality status of the connected biobanks, to define the areas of improvement within the biobanks and to develop tailored support by the Quality working group. To this end, we launched 2 online surveys (fall 2017 and fall 2018) to the biobank quality managers (12 and 13, respectively) in the BBMRI.be network to determine the status and setup of their current QMS and how their QMS and related practices have evolved over a 14 month time period.

## Materials and Methods

### First Survey

Fall 2017, a short, high level questionnaire made using the SurveyMonkey™ tool was distributed to the quality managers of biobanks linked to BBMRI.be to assess the general status and activities with respect to the QMS. The survey was distributed to the 12 biobanks of the BBMRI.be network, by providing a link in an explanatory email. For those biobanks which did not have a separate quality manager (e.g., due to limited biobank staff size), the general biobank manager was addressed. The survey questions are available in the Supplementary Material.

### Second Quality Survey

The second, more detailed Quality survey was designed using REDCap electronic data capture tools hosted at Ghent University Hospital. REDCap (Research Electronic Data Capture) (20) is a secure, web-based application designed to support data capture for research studies, providing: (1) an intuitive interface for validated data entry; (2) audit trails for tracking data manipulation and export procedures; (3) automated export procedures for seamless data downloads to common statistical packages; and (4) procedures for importing data from external sources. The survey was distributed to the 13 (2018) biobanks of the BBMRI.be network, by providing a link in an explanatory email. The survey consisted of a dynamical questionnaire, visualizing additional questions dependent on the responses given to deepen the answers given to the core set of questions. The content focused on three main sections. The first section captured the general information of the biobank and the QMS system. The second part focused on the specific procedures present in the QMS system and the supportive systems used. The third part addressed the needs related to Quality of the BBMRI.be biobank community. The survey questions are available in the Supplementary Material.

### Survey Analysis

The survey data of both surveys was exported into a spreadsheet and data analysis was performed using the REDCap data analysis tool combined with Sigmaplot for graph design.

## Results

### Properties of BBMRI.be Biobanks

**Figure 1:**
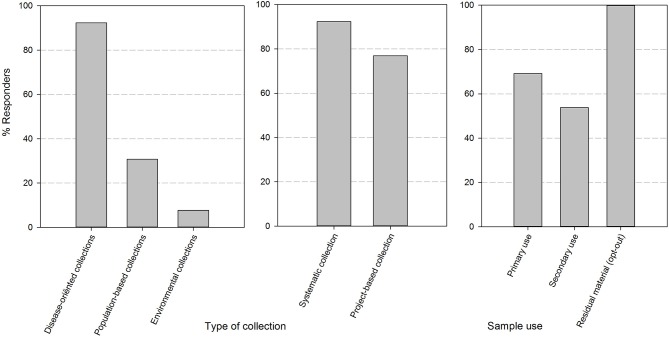
The set-up and composition of human body material collections in biobanks of the BBMRI.be network. Responses were divided by the nature of the human body material collection (collection with a disease orientation, population-based or on environmental ground), by the method of collection of the material (systematic i.e., by sampling at occasions within the clinical path of the patient or project-based i.e., by sampling in the framework of a specific study/project at pre-defined and study-based time points) and by the type of use as defined in the Belgian legislation (primary use: the use to which the donor has explicitly consented to at the time of collection; secondary use: any other use than the one consented to at the time of collection; residuary material: the portion of human bodily material that has been taken from the donor for diagnostic or treatment purposes which, when a sufficient sample is safeguarded for refinement or completion of these purposes, is superfluous to these purposes and as such could be destroyed—presumed consent applies).

After sending out the surveys, all quality/biobank managers (respectively, 12/12 in 2017 and 13/13 in 2018) targeted in the mailing submitted a complete set of responses for the two online questionnaires. The majority of biobanks were university hospital integrated (9/13). The responders also included 1 academic biobank, 1 general hospital-based biobank and 2 biobanks of research institutions (type non-profit organization or association without lucrative purpose). The responders are biobanks with multiple types of collections in their catalog, as can be seen in Figure 1. These collections are mainly disease-oriented (92%) and originate from both systematic (92%) and project/study based (77%) approaches to sample collection. All of the biobanks collect residuary material cleared by presumed consent, in compliance to current Belgian legislation. In addition, 69% of biobanks collect samples for primary use and 54% of biobanks also distribute samples for secondary use (defined by Belgian law as any use different from that to which was consented by the donor at the time of collection of the specimen).

Of the 13 responders, 8 biobanks are part of a certified/accredited lab environment (60% ISO 15189, 10% ISO 17025). Three biobanks had obtained an ISO 9001 certificate specifically for their biobanking activities. Sixty two percent of the responders receive samples of an accredited lab environment [Pathology lab (ISO 15189), Clinical-Analytic lab (ISO 17025) or Medical Genetics lab (ISO 15189) or JACIE accredited facility].

### QMS Status and Sources of Belgian Biobanks

**Figure 2:**
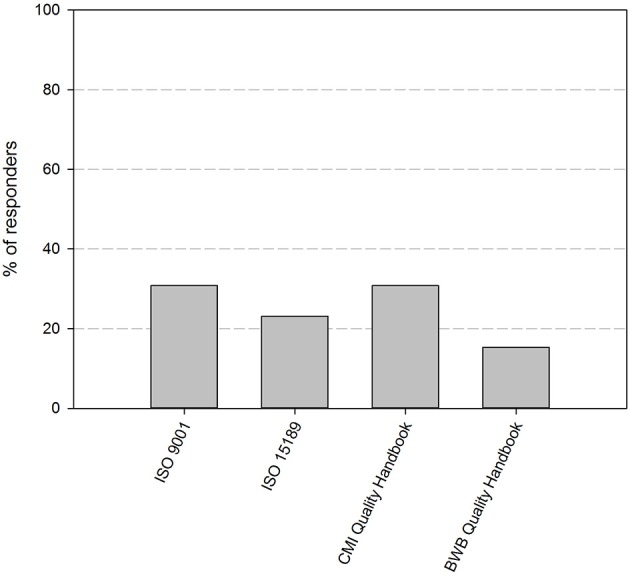
Quality standards or guidelines used as primary basis for the quality management system of the biobanks in the BBMRI.be network. Participants were asked to select the standard or guideline used as primary basis for their QMS from a list of nine standards/guidelines (ISO 9001, ISO 15189, ISO 17025, ISO 20387, CMI QMS Guidelines, BWB QMS Guidelines, ISBER Best Practices, OECD Recommendations for Biorepositories, French Biobanking standard NF S96-900). Only one option could be selected. Only four standards/guidelines were indicated by the responders to be used as primary basis for QMS systems, as displayed in the figure. QMS, quality management system.

At the time of the first survey in the fall of 2017, 11 out of 12 participating biobanks had implemented a QMS. By the time of the second survey, about 1 year later, 85% of the biobanks have implemented an operational QMS system. The remaining 15% of responders is currently in the process of implementing a QMS system. Four guidelines stand out as primary basis for the QMS: ISO 9001 (31%), CMI quality guidelines (31%), ISO 15189 (23%), and the BWB quality guidelines (15%) (Figure 2). Apart from one biobank, all biobanks use additional guidelines for their QMS (Table 1). The most frequently used are the ISBER guidelines for biobanks (69%), the ISO 9001 standard (67%), and the OECD guidelines for biorepositories (54%). Fifty percent of responders applied the Standard Pre-analytical Code, either automatically (33%), or manually (17%), while the other half intended to implement SPREC in the future. Only 1 responder had implemented BRISQ and only 1 responder intended to implement it in the future (data not shown).

**Table 1:** Primary and secondary QMS standards and guidelines used in Belgian biobanks.

**Responder**	**ISO 9001**	**ISO 15189**	**ISO 17025**	**ISO 20387**	**CMI QMS guidelines**	**BWB QMS guidelines**	**ISBER**	**OECD**	**NF S96-900**
1	Prim	Sec	–	Sec	–	Sec	Sec	Sec	–
2	Prim	Sec	Sec	Sec	Sec	–	Sec	Sec	Sec
3	Prim	–	–	–	–	–	–	–	–
4	Prim	–	–	–	–	Sec	–	–	–
5	Sec	Prim	Sec	–	Sec	–	Sec	Sec	–
6	Sec	Prim	–	Sec	–	Sec	Sec	Sec	Sec
7	–	Prim	–	–	–	Sec	Sec	Sec	Sec
8	Sec	–	–	–	Prim	–	–	–	–
9	Sec	–	–	Sec	Prim	–	Sec	–	–
10	Sec	Sec	–	Sec	Prim	–	Sec	Sec	Sec
11	–	Sec	Sec	–	Prim	–	Sec	Sec	–
12	–	–	–	–	–	Prim	Sec	–	–
13	Sec	–	–	–	-	Prim	–	–	–
Total # of QMS standard use	10	7	3	5	6	6	9	7	4
# of secondary QMS standard use	6	4	3	5	2	4	9	7	4

*Prim, standard/guideline used as primary basis for QMS; Sec, standard/guideline used as supplementary basis for QMS; QMS, Quality Management System*.

**Figure 3:**
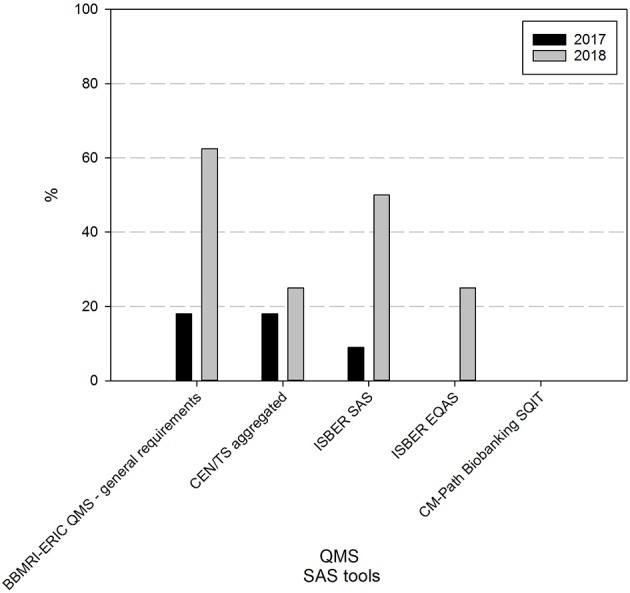
Use of self-assessment tools in biobanks in the BBMRI.be network in 2017 and 2018. Participants were asked to indicate from a list which self-assessment survey (SAS) tools were used in their biobank to assess the status of the overall quality management system (BBMRI-ERIC QMS—general requirements and ISBER SAS), the compliance to standard specifications for pre-examination processes (CEN/TS aggregated: 10 individual SAS tools developed by BBMRI-ERIC, participation in one or more of these SAS tools was considered as 1 positive reply), the overall quality status of the pre-analytical processes (ISBER EQAS) or awareness of factors in tissue collection, processing and storage that may impact sample quality (CM Path Biobanking SQIT). Black bars indicate participants' responses in 2017, gray bars indicate participants' responses in 2018. CEN/TS, European Committee for Standardization Technical Standards; EQAS, pre-analytical external quality assessment survey; SQIT, CM-Path Biobanking Sample Quality Improvement Tool.

At the time of the first survey, a majority (73%) had never taken part in freely available online self-assessment surveys (SAS) (9% had taken the ISBER SAS, 18% the BBMRI-ERIC general QMS SAS or the BBMRI-ERIC SAS to check compliance to the CEN technical standards for pre-examination processing of biospecimens). One year on, 62% of responders are using one or more self-assessment tools. The BBMRI-ERIC general QMS SAS (63%) and the ISBER SAS (50%) are the most commonly applied tools in the BBMRI.be network (Figure 3).

Fall 2018, 54% of the responders was participating in yearly external proficiency testing for their testing or processing methods. Of these responders, 71% is using biobank specific proficiency testing programs (such as the IBBL proficiency testing program), which is an increase compared to the number reported the year before (46%). Biobanks embedded in accredited laboratories can participate in laboratory related proficiency schemes and 43% of the biobanks make use of this possibility (data not shown). Several reasons were given for not participating in the biobank specific proficiency testing schemes: (i) it is not requested by the providers or customers (4/6), (ii) the high cost of participation (2/6), (iii) the lack of added value for the biobank (1/6), and (iv) the adequacy of available biobank testing schemes (1/6).

### Status of QMS and Biobank Requirements in BBMRI.be Biobanks

**Figure 4:**
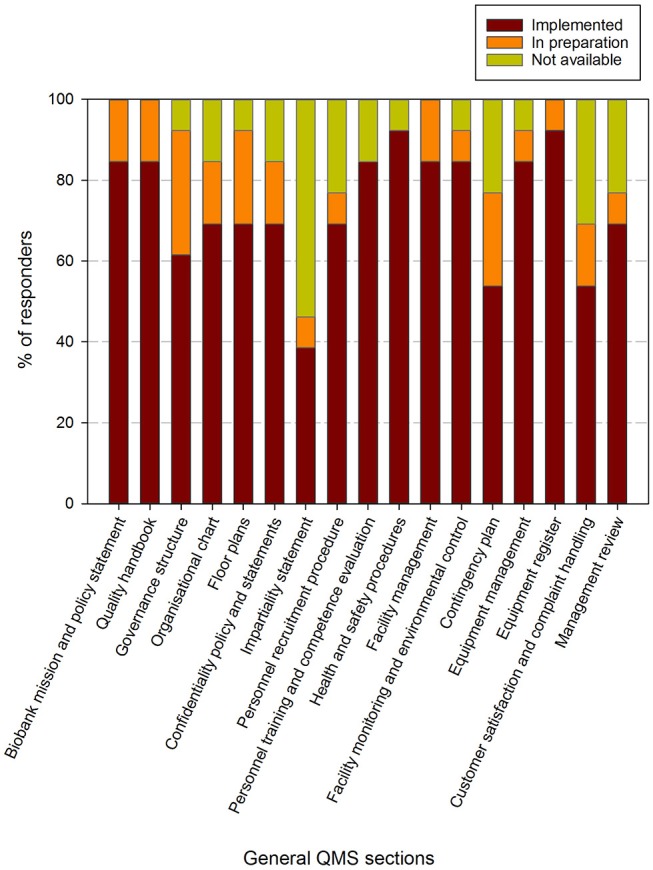
Implementation status of general procedures in the quality management system in biobanks in the BBMRI.be network. Participants were requested to indicate for each item in a list of general procedures/items whether these were fully implemented (brown), in preparation (orange) or not available (lime-green). The items listed were collected and aggregated from the ISBER best practices for biorepositories, the ISO 20387 and ISO 9001 standards. All participants responded to all items listed. The list of items asked is displayed in the X-axis.

**Figure 5:**
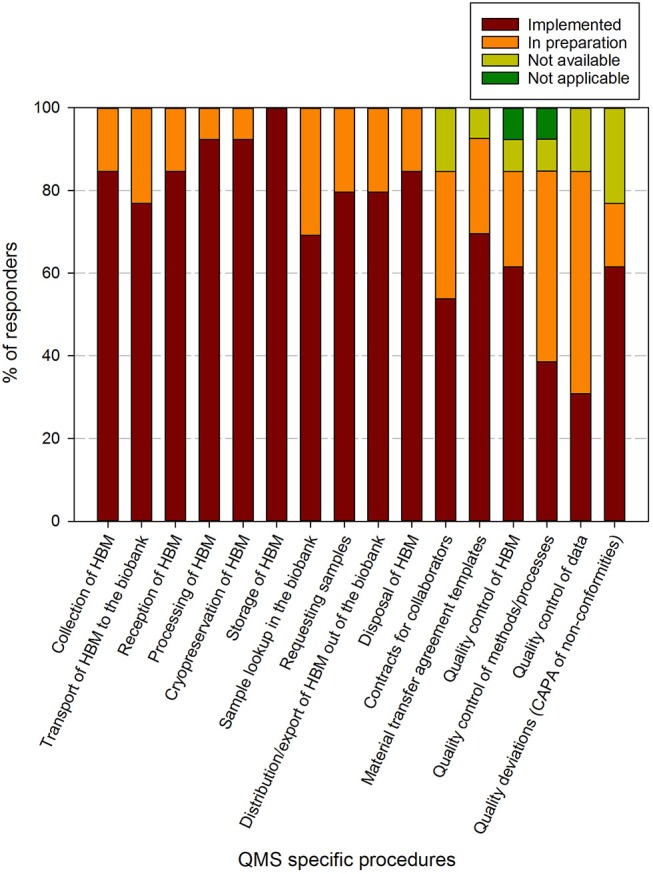
Implementation status of biobank specific procedures in biobanks in the BBMRI.be network. Participants were requested to indicate for each item in a list of specific procedures/items whether these were fully implemented (brown), in preparation (orange), not available (lime-green), or not applicable (dark green). The items listed were collected and aggregated from the ISBER best practices for biorepositories, the ISO 20387 and ISO 9001 standards. All participants responded to all items listed. The list of items asked is displayed in the X-axis.

In the questionnaire, an aggregated list of required procedures for ISO 9001:2015, ISO 20387:2018, and the ISBER best practices (fourth edition) was presented to the participants. An overview of the responses regarding the general and biobank specific procedures and requirements is shown in Figures 4, 5, respectively.

Eight out of 17 general QMS requirements are fulfilled by more than 80% of responders and an additional 6 have been implemented by 60–80% of responders. Two items have been implemented by 40–60% of responders (i.e., contingency plan and customer satisfaction & compliant handling) and 1 item was available in <40% of responding biobanks (i.e., impartiality statement). When also taking the responders into account where the procedures were in preparation, 12 out of 17 requirements were being addressed by over 80% of responders. Again the impartiality statement was the least addressed, whereas the contingency plan, customer satisfaction and complaint handling, personnel recruitment and management review were the procedures that were proportionally less present (Figure 4).

Regarding the biobank specific requirements, 8 out of 16 items had been implemented by >80% of responders at the time of the survey, 5 by 60–80%, 1 by 40–60% (contracts for collaborators) and 2 by 20–40% (quality control of methods/processes and quality control of data). The procedure for storage of human bodily material was present in all biobanks. When taking into account the responders that are in implementation phase for the procedures, 16 out of 17 items were being addressed in more than 80% of biobank QMS. The remaining item, quality deviations or corrective/preventive actions, was reported in 60–80% of responders (Figure 5). One biobank indicated that quality control of human bodily material or quality control of methods/processes was not applicable for their activities.

**Figure 6:**
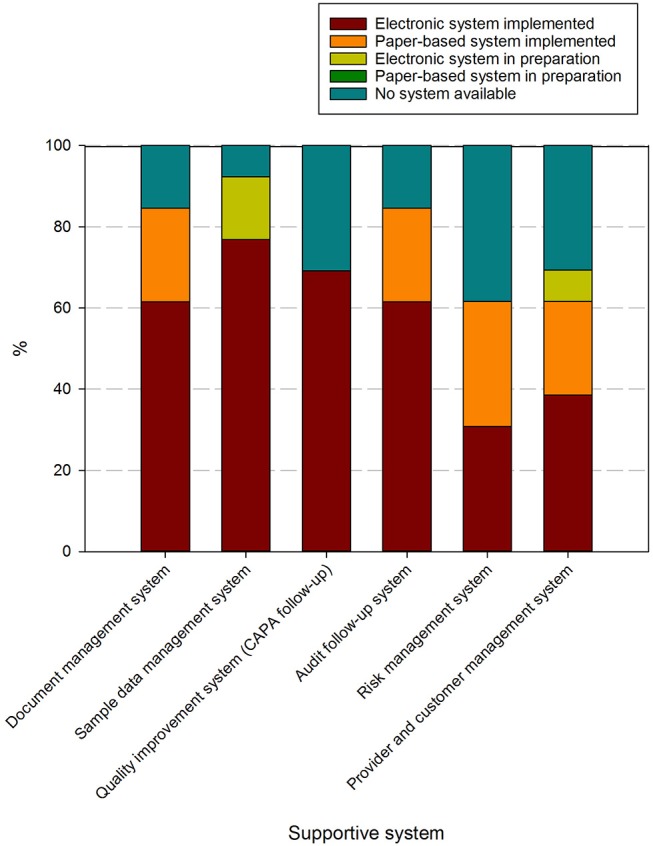
Overview of systems used to support activities in biobanks in the BBMRI.be network. Participants were asked to indicate in a list for which supportive tools for the QMS an electronic system was implemented (brown), a paper-based system was implemented (orange), an electronic system was in preparation (lime-green), a paper-based system was in preparation (dark green), or no system was available (petrol blue). All participants responded to all items listed. The list of items asked is displayed in the X-axis.

Supportive systems used to handle these processes contained in these procedures are present in at least 60% of responders, although only document management and audit follow up systems are used at over 80% of biobanks (Figure 6). Risk management systems and provider/customer management systems are the least available. Predominantly electronic/digital systems are utilized, although paper-based systems still occur for document management, audit follow-up, risk management and provider/customer management.

### Future Goals and Needs of the Belgian Biobanks

**Figure 7:**
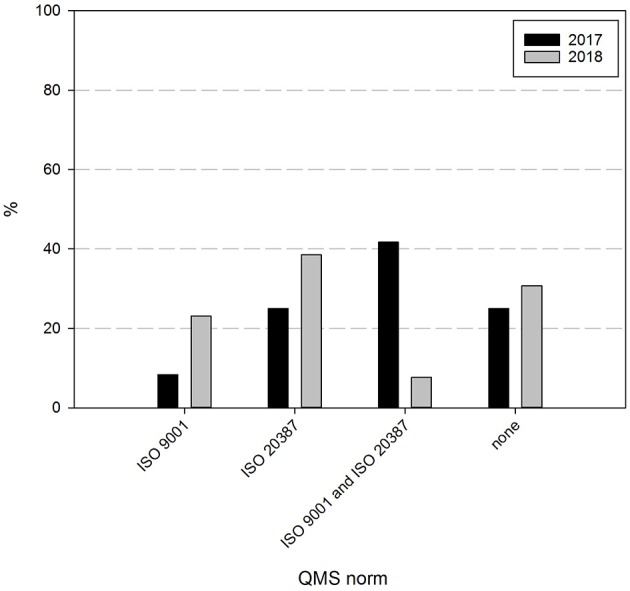
Certification intentions biobanks in the BBMRI.be network. Participants were asked to indicate which if any certification/accreditation they intended to acquire within 2 years. Black bars show participants' responses in 2017, gray bars show participants' responses in 2018. All participants responded to all items listed.

Finally, the intentions and perspectives of Belgian biobanks were assessed. Sixty nine percent of the responders indicate to strive for biobank certification and/or accreditation within 2 years, as can be seen in Figure 7. Although the percentage of biobanks intending to acquire certification is similar to the one indicated the year before (75%), the intended certification has shifted. In 2017, 42% of biobanks aimed for both ISO 9001 and ISO 20387 certification, 25% for ISO 20387 and 8% for ISO 9001 certification, while in 2018 this has focused to nearly 38% intending ISO 20387 certification, 23% ISO 9001, and only 8% of responders still intending to acquire both ISO 9001 and ISO 20387 certification. About 31% of responders did not intend to acquire certification.

The majority of responders (77%) indicated a clear need for a Belgian SAS tool, adapted to the national biobank law which has come into effect on November 1st 2018. The need for a national proficiency program, on top of already existing schemes, was put forward by 54% of the responders (data not shown).

An overwhelming majority (12/13) of the responders expressed a need for the setup of a national peer-review audit program. Several types of peer-review audit programs were suggested: 75% of the responders would prefer a two-phase audit, consisting of an initial administrative/documentary audit (off-site) of the biobank procedures followed by a site visit 1 year later. Twenty five percent of the responders would prefer a full on-site audit straightaway.

## Discussion

Irreproducibility of results, also originating from the biospecimens used, has been identified as a major undermining factor for translating research results into clinical applications. Biobanks can play an important role by providing fit-for-purpose (human) bodily material, governed through a professional QMS according to evidence-based guidelines and/or standards. Since 2013, the BBMRI.be network brings together the large academic/non-profit research biobanks in Belgium and strives through working groups for harmonization on multiple levels. The BBMRI.be Quality working group setting new goals in alignment with BBMRI-ERIC to further professionalize qualitative biobanking in Belgium. To this end, the Quality working group performed two surveys over the course of a year to gauge the current quality status of the connected biobanks, to define the areas of improvement in the biobanks themselves and in the support delivered by the Quality working group.

The surveys were targeted to the Quality managers of the biobanks or in absence of a dedicated person due to biobank size, to the overall operational manager of the biobank. One completed survey response was requested and received per biobank. The biobanks show a big diversity in nature and type of their collections. In the Belgian biobank legislation, residual material can be obtained and used for research via an opt out system (21), and a third of the BBMRI.be biobanks consist entirely of residuary material collections. The use of residuary material is not the default situation within the global biobanking community, but it can show an impressive track record of valuable results, provided certain quality and ethical conditions are met (22). The results of this survey mainly reflect the activities of academic research biobanks, which are currently the main members of BBMRI.be. However, BBMRI.be intends to represent the complete Belgian biobank landscape by also reaching out to other institutional and/or commercial/private biobanks in the near future, in an effort to address commonly encountered biobank related issues. Uniting the Belgian biobanks through BBMRI.be may also further improve and harmonize biospecimen quality and consequentially contribute to an increased reproducibility of translational research.

The survey results show that the BBMRI.be related biobanks have a highly developed “quality” mindset, as the grand majority of the biobanks have implemented a formal QMS and the remaining biobanks are in progress of implementing a system. The primary basis of the implemented QMS system is different between the responders. This divergence has both historical and organizational reasons. The FBN and the BWB were already active and had published their quality handbooks before the BBMRI.be community was set up. Additionally, biobanks that are integrated in a ISO 15189 or ISO 17025 accredited laboratory prefer to take this standard as primary basis of their QMS system. Even so, all of the aforementioned guidelines/standards used as a primary basis have a direct reference to ISO 9001, the commonly accepted standard for general quality management systems, indicating that the Belgian biobank QMS's contain a similar general basis for their activities and procedures.

The majority of biobanks take additional norms/guidelines into account to develop their QMS. The variety of widely available biobank guidelines is mirrored in the responses of the participants, with the ISBER guidelines, the OECD guidelines and ISO 9001 being the most popular. This diversity is likely caused by the initial absence of a relevant international norm covering all of the activities of a biobank (23), a gap recently filled by the biobank centered ISO 20387 standard (24). Despite its recent publication, it is already being picked up by the biobanks in the BBMRI.be network. However, the ISO 20387 standard was developed to be complementary to and to be used with the existing biobank guidelines from the start to strengthen a biobank's pursuits for quality management (25). This allows biobanks to tailor their own needs but also leaves room for deviation based on the guidelines used.

The majority of procedures for general and biobanking specific activities, aggregated from the ISBER Best Practices and the ISO 9001 and ISO 20387 standards, were present in the BBMRI.be connected biobanks. The items with the lowest compliance rates were either requirements from the recently published biobanking ISO standard (e.g., impartiality statement) or items that had received more focus due to the publication of the standard (e.g., quality control of biospecimens and/or methods/processes). The latter however are actively being implemented by the non-compliant biobanks, indicating that these have gained importance in the biobank activities. In comparison to the results of the ISBER self-assessment participants, the BBMRI.be biobanks score better at several of the commonly asked items, both general and biobank specific, again emphasizing the quality-mindset within the participants (26).

We acknowledge that the results reported in this study are self-reported and may therefore overestimate the actual status of the responders' QMS. However, the lagging of the business aspects of biobanking is in line with data from a recently performed international study on biobank business operations (27). Although this part of biobanking is an important factor for success, it is a known gap in the community and the subject has recently gained more visibility to professionalize this side of biobank operations (28). Given the raised awareness regarding this aspect in literature and biobank standards, we expect to see progress in this area in future surveys.

Self-assessment tests and participation in proficiency testing schemes are recognized ways to monitor the QMS controlled activities and define areas for improvement. Initially these tools were not very well-known within the biobanks of BBMRI.be, but their participation rate has greatly increased over the 14 month period covered by the surveys. One factor explaining this success might be the indirect education through the surveys and the presented first survey results.

Although most of the BBMRI.be biobanks indicate to use the ISBER Best Practices as inspiration for their QMS, only half of them have already taken the ISBER self-assessment survey. Still, this is an increased proportion compared to the 62 global biobanks that completed the full survey in the period 2015–2017 (26). The biobanks might consider the self-assessment as a premature activity, since some of the essential biobank processes might still be in the implementation phase. Additionally, diagnostic self-assessment surveys have lost a bit of their appeal, with external audits gaining a more apparent value. This is also reflected by the fact that nearly all participants were in favor of setting up a national peer-review audit program. Similar audit initiatives have been and are being set-up in different national biobank networks (29, 30), emphasizing the need felt by biobanks to comply with the audit requirements stated by guidelines and standards. Although these within-network audits have the advantage of allowing to assess local legislative and regulatory requirements, it may also introduce quality differences between these networks. The European research infrastructure BBMRI-ERIC therefore intends to setup a peer-review audit program framework to be used by the member states, leaving room for local peculiarities while maintaining an independent comparative evaluation.

Peer-review audits can also serve as preparation for intended certification or accreditation activities. With about three quarter of participants intending to acquire certification for ISO 9001, ISO 20387, or both within 2 years, the implementation of a peer-review audit program might support the BBMRI.be biobanks in achieving this goal. This intention is in line with the ongoing evolution to “biobanking 3.0,” with an increased focus on operational standardization of processes (31). A key element initially unavailable to biobanks in this respect has been quality assessment by an external organization (32). Two currently available international programs show a high overall success rate (33, 34). Furthermore, the new standard ISO 20387 will allow biobanks to pursue accreditation or certification for their activities, formalizing their competence (25). The recent publication of this standard is also likely to explain our observed shift of the combined ISO 9001 & ISO 20387 intention toward the majority opting for ISO 20387 in the second survey.

The participants expressed a clear need for a national peer review program and a self-assessment survey fit to the Belgian legislation. These requests can be addressed by the BBMRI.be Quality working group, by developing add-ons to existing or starting international initiatives in order to harmonize to the global community. Given the resources available, the initial focus will be put on the implementation of the audit program, building on the FBN peer review audit. About half of the responders indicated a need for a national proficiency testing scheme. It has been shown that repeated participation in biobank proficiency schemes can indeed lead to global improvement of performance (35, 36). However, about half of the responders are currently not participating in already available proficiency schemes. It is therefore opted by Quality working group to educate the biobanks regarding the existence and advantage of proficiency testing programs as a more valuable first step.

Overall, the biobanks of the BBMRI.be network have actively implemented a quality approach in their daily practices, though room for improvement exists. The implementation of ISO20387 may bring further professionalization of activities. Based on the current needs expressed in this survey, the Quality working group will be setting up a novel audit program for the BBMRI.be biobanks, to enhance, harmonize and streamline activities. Additionally, raising further awareness about self-assessment tools that are freely available, proficiency testing schemes and the value of performing these tests will be on the agenda in the coming months and years. On the whole, the biobanks in the BBMRI.be network are able to contribute to better translational research through a sustained quality approach.

## Data Availability

All datasets generated for this study are included in the manuscript and/or the Supplementary Files.

## Author Contributions

LL, VT, CV, KV, EM, SB, and NE conceived of the presented idea. LL and VT designed the surveys, analysed the results and wrote the article. All authors critically reviewed the manuscript.

### Conflict of Interest Statement

The authors declare that the research was conducted in the absence of any commercial or financial relationships that could be construed as a potential conflict of interest.

## References

[1] Begley CG Ellis LM. Drug development: raise standards for preclinical cancer research. Nature. (2012) 483:531–3. 10.1038/483531a 22460880

[2] Freedman LP Cockburn IM Simcoe TS. The economics of reproducibility in preclinical research. PLoS Biol. (2015) 13:1–9. 10.1371/journal.pbio.1002165 26057340 PMC4461318

[3] Furman JL Stern S Climbing atop the shoulders of giants: the impact of institutions on cumulative research. Am Econ Rev. (2011) 101:1933–63. 10.1257/aer.101.5.1933

[4] Campbell LD Astrin JJ DeSouza Y Giri J Patel AA Rawley-Payne M. The 2018 revision of the ISBER best practices: summary of changes and the editorial team's development process. Biopreserv Biobank. (2018) 16:3–6. 10.1089/bio.2018.0001 29393664 PMC5846567

[5] OECD Best Practice Guidelines for Biological Resource Centres [Internet]. (2007). Available online at: http://www.oecd.org/sti/emerging-tech/38778261.pdf (accessed March 22, 2019)

[6] OECD Guidelines on Human Biobanks and Genetic Research Databases [Internet]. (2009). Available online at: http://www.oecd.org/science/emerging-tech/44054609.pdf (accessed March 22, 2019)

[7] Schacter B Mes-Masson A-M Damaraju S Albert M de Sousa-Hitzler J Watson P. Generating a comprehensive set of standard operating procedures for a biorepository network—The CTRNet experience. Biopreserv Biobank. (2013) 11:387–96. 10.1089/bio.2013.0061 24835369

[8] WHO/IARC Common Minimum Technical Standards and Protocols for Biobanks Dedicated to Cancer Research [Internet]. (2017). Available online at: http://publications.iarc.fr/551 (accessed March 22, 2019) 33539055

[9] Rao A Vaught J Guan P Weil C Moore H The U.S. National Cancer Institute's 2016 best practices for biospecimen resources. Biopreserv Biobank. (2017) 15:60.

[10] Dagher G Becker K Bonin S Foy C Gelmini S Kubista M. Pre-analytical processes in medical diagnostics: new regulatory requirements and standards. N Biotechnol. (2019). 10.1016/j.nbt.2019.05.002. [Epub ahead of print]. 31102798

[11] Heinrich M Oelmueller U Krieger L Riegman P Becker KF Pazzagli M SP036 SPIDIA – dissemination of results into CEN technical specifications for biospecimen handling. Eur J Cancer. (2013) 49:S10 10.1016/S0959-8049(13)70114-X

[12] Betsou F Bilbao R Case J Chuaqui R Clements JA De Souza Y Standard PREanalytical code version 3.0. Biopreserv Biobank. (2018) 16:9–12. 10.1089/bio.2017.0109 PMC11708182 29377712

[13] Betsou F Lehmann S Ashton G Barnes M Benson EE Coppola D. Standard preanalytical coding for biospecimens; defining the sample PREanalytical code. Cancer Epidemiol Biomarkers Prev. (2010) 19:1004–11. 10.1158/1055-9965.EPI-09-1268 20332280

[14] Norlin L Fransson MN Eriksson M Merino-Martinez R Anderberg M Kurtovic S. A minimum data set for sharing biobank samples, information, and data: MIABIS. Biopreserv Biobank. (2012) 10:343–8. 10.1089/bio.2012.0003 24849882

[15] Moore HM Kelly AB Jewell SD McShane LM Clark DP Greenspan R Biospecimen reporting for improved study quality (BRISQ). J Proteome Res. (2011) 8:3429–38. 10.1021/pr200021n PMC3169291 21574648

[16] Merino-Martinez R Norlin L Van Enckevort D Anton G Schuffenhauer S Silander K. Toward global biobank integration by implementation of the minimum information about biobank data sharing (MIABIS 2.0 Core). Biopreserv Biobank. (2016) 14:298–306. 10.1089/bio.2015.0070 26977825

[17] Groningen R Fundamentals of Biobanking and Cohort Research [Internet]. Available online at: https://www.rug.nl/research/gradschool-medical-sciences/summerschools/biobanking/ (accessed March 22, 2019)

[18] Luxembourg U of Certificate - Principles of Biobanking [Internet]. Available online at: https://wwwfr.uni.lu/formations/fstc/certificate_principles_of_biobanking (accessed March 22, 2019)

[19] Gormally E di Donato J-H Caboux E Hofman P Hainaut P Hardy I. Training the next generation of biobankers: a two-year master's course in the management of biobanks. Biopreserv Biobank. (2017) 15:438–50. 10.1089/bio.2017.0002 28922617

[20] Harris PA Taylor R Thielke R Payne J Gonzalez N Conde JG. Research electronic data capture (REDCap)-A metadata-driven methodology and workflow process for providing translational research informatics support. J Biomed Inform. (2009) 42:377–81. 10.1016/j.jbi.2008.08.010 18929686 PMC2700030

[21] Law Regarding Acquisition and Use of Human Bodily Material for Therapeutic Purposes and Scientific Research (Dutch) [Internet]. (2008). Available online at: http://www.ejustice.just.fgov.be/cgi_loi/change_lg.pl?language=nl&la=N&cn=2008121944&table_name=wet (accessed March 22, 2019)

[22] Riegman PHJ Van Veen EB. Biobanking residual tissues. Human Genetics. (2011) 130:357–68. 10.1007/s00439-011-1074-x 21814798

[23] Betsou F Luzergues A Carter A Geary P Riegman P Clark B Towards norms for accreditation of biobanks for human health and medical research: compilation of existing guidelines into an ISO certification/accreditation norm-compatible format. Qual Assur J. (2007) 11:221–294. 10.1002/qaj.425

[24] International Organization for Standardization ISO 20387:2018 Biotechnology – Biobanking – General Requirements for Biobanking [Internet]. (2018). Available online at: https://www.iso.org/standard/67888.html (accessed March 22, 2019)

[25] Furuta K Allocca CM Schacter B Bledsoe MJ Ramirez NC. Standardization and innovation in paving a path to a better future: an update of activities in ISO/TC276/WG2 biobanks and bioresources. Biopreserv Biobank. (2018) 16:23–7. 10.1089/bio.2017.0117 29394084

[26] Betsou F. The ISBER self-assessment tool indicates main pathways for improvement in biobanks and supports international standardization. Biopreserv Biobank. (2018) 16:7–8. 10.1089/bio.2017.0121 29359960

[27] Henderson MK Goldring K Simeon-Dubach D. Advancing professionalization of biobank business operations: a worldwide survey. Biopreserv Biobank. (2018) 17:71–5. 10.1089/bio.2018.0079 30412417 PMC6389761

[28] Vaught J Kelly A Hewitt R. A review of international biobanks and networks: success factors and key benchmarks. Biopreserv Biobank. (2010) 7:143–50. 10.1089/bio.2010.0003 24835880 PMC4046743

[29] The UKCRC Tissue Directory and Coordination Centre The CCB's Pilot Audit Scheme [Internet]. Available online at: https://www.biobankinguk.org/accreditation/ (accessed March 22, 2019)

[30] BBMRI-ERIC BBMRI-ERIC QUALITY MANAGEMENT SERVICES - Auditing [Internet]. Available online at: http://www.bbmri-eric.eu/services/quality-management/ (accessed March 22, 2019)

[31] Simeon-Dubach D Watson P. Biobanking 3.0: evidence based and customer focused biobanking. Clin Biochem. (2014) 47:300–8. 10.1016/j.clinbiochem.2013.12.018 24406300

[32] Carter A Betsou F. Quality assurance in cancer biobanking. Biopreserv Biobank. (2011) 9:157–63. 10.1089/bio.2010.0031 24846261

[33] Dash RC Robb JA Rose J Harrison JH Dry SM Mateski DL. The college of american pathologists biorepository accreditation program: results from the first 5 years. Biopreserv Biobank. (2018) 16:16–22. 10.1089/bio.2017.0108 29394087 PMC5824654

[34] Barnes RO Shea KE Watson PH. The canadian tissue repository network biobank certification and the college of american pathologists biorepository accreditation programs: two strategies for knowledge dissemination in biobanking. Biopreserv Biobank. (2016) 15:9–16. 10.1089/bio.2016.0021 27740852

[35] Gaignaux A Ashton G Coppola D De Souza Y De Wilde A Eliason J. A biospecimen proficiency testing program for biobank accreditation: four years of experience. Biopreserv Biobank. (2016) 14:429–39. 10.1089/bio.2015.0108 27195612 PMC6445200

[36] Biorepository proficiency testing for the quality control of biospecimens for the global biobanking community Biopreserv Biobank. (2011) 9:415–7. 10.1089/bio.2011.9402 24836637

